# ReFaceX: donor-driven reversible face anonymisation with detached recovery

**DOI:** 10.1038/s41598-026-39337-2

**Published:** 2026-02-09

**Authors:** Dost Muhammad, Muhammad Salman, Syed Muhammad Haider Shah, Malika Bendechache

**Affiliations:** 1 Research Ireland Center of Research Training and Artificial Intelligence (CRT-AI), Galway, Ireland; 2 Department of Software Engineering, University of Malakand, Malakand, Pakistan; 3 ADAPT Research Centre, School of Computer Science, University of Galway, Galway, Ireland; 4 School of Computer Science, University of Galway, Galway, Ireland

**Keywords:** Deep learning in imaging, ReFaceX, Reversible face anonymisation, Donor-driven identity transfer, Identity feature fusion, Image steganography, Detached recovery, Privacy utility trade off, Open set re-identification, Face recognition auditing, Engineering, Mathematics and computing

## Abstract

Organisations must share facial imagery that remains useful for analysis while protecting identity. Many current methods fail to strike this balance: reconstruction-centred encoder–decoder designs tend to blur salient detail, whereas latent edits in pretrained generators often retain or drift identity cues, undermining privacy and utility. We present ReFaceX, a reversible anonymisation framework that separates what to protect from what to preserve. A donor identity code steers a U-Net anonymiser with Identity Feature Fusion to change identity while retaining non-identity content such as pose, background and expression. A learned steganographic channel carries a compact recovery payload, and reconstruction gradients are blocked at the stego image so the anonymiser is never rewarded for keeping identity. The threat model is stated explicitly and outcomes are audited with strong recognisers. On LFW and CelebA-HQ datasets at $256\times 256$, ReFaceX reduces identity similarity across FaceNet, ArcFace and AdaFace, and improves recovered-image quality (SSIM $0.9378$, LPIPS $0.1002$, PSNR $23.97$ dB), while operating in real time on a single RTX 3090. Robustness to common JPEG re-encoding is also demonstrated. By turning the privacy–utility balance into an explicit and auditable operating choice, ReFaceX provides a practical template for responsible release of facial imagery and a foundation for extensions to video, higher resolutions and stronger recovery guarantees.

## Introduction

**Fig. 1:**
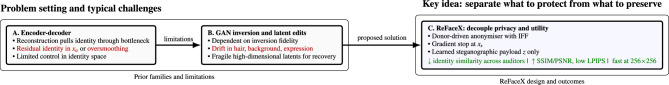
Motivation and problem setting. Left: encoder-decoder anonymisers often retain identity or blur detail; GAN inversion can alter non-identity attributes and is fragile to real transforms. Right: ReFaceX separates privacy from utility using donor-driven IFF, a gradient stop at $x_s$, and a learned steganographic payload. Outcome: lower identity similarity under multiple auditors with high recovered-image fidelity and fast inference.

Public release and reuse of facial imagery in science, industry and government require anonymisation that protects identity while preserving analytical utility for tasks such as detection, tracking and landmark localisation. Naive obfuscation by blurring or mosaicing often destroys salient cues and still leaks identity to modern recognisers^1–4^. This has motivated a wave of deep learning methods that synthesise identity-altered faces with higher visual fidelity^2,5,6^, and a subset of reversible approaches that aim to allow authorised recovery of the original content^7–9^. A compact visual summary of these challenges and the problem setting is provided in Fig. 1.

Despite considerable progress, three limitations recur across existing lines of work. First, the security model is often implicit. Many methods optimise anonymisation against surrogate recognition losses and report verification-style metrics, yet they do not fix a concrete adversary with explicit knowledge, data and compute, nor do they provide guarantees such as bounds on identity leakage^3,4^. Reversible designs that store a recovery key or code within the anonymised image enlarge the attack surface unless the channel is robust to realistic distortions and resistant to extraction by an adaptive adversary^7^. Secondly, recovery channels are rarely stress-tested. In realistic deployments anonymised images are re-encoded, resized, cropped and filtered by social platforms. Without batteries that cover JPEG at multiple qualities, rescaling, cropping, colour space changes and codec transcodes, reliability outside the laboratory remains unclear^10^. Thirdly, empirical scope is narrow. Encoder-decoder anonymisers tend to favour over-smooth reconstructions^5,6^, while latent-space manipulation and inversion in pretrained GANs can reduce detail or shift attributes when inversion is imperfect^11,12^. Subgroup performance across age, skin tone and gender is rarely reported, which limits claims about fairness, and almost all results are image based with aligned crops rather than video with temporal consistency.

We present ReFaceX as a practical integration of established components configured to address these gaps under a clear threat model. Identity modification is steered by a donor identity embedding injected into a U-Net backbone through an identity feature fusion block^13^, so that identity change is large and controlled while non-identity content is preserved. Recovery is optimised on a detached computational path, which prevents the anonymiser from retaining identity cues merely to ease reconstruction. A compact recovery code is carried by a learned steganographic channel with an explicit objective that forces the encoder-decoder pair to transport information rather than allowing a bypass through near-identity outputs^7^. A high-level contrast with prior families and the core idea of separating privacy from utility are illustrated in Fig. 1. We do not claim these ingredients to be new in isolation; the contribution lies in decoupling privacy from utility during training, auditing outcomes against strong recognisers, and stress-testing the recovery channel under realistic distortions.

We formalise a threat model spanning black-box and white-box adversaries built around strong face recognition encoders^3,4^ and open-set search. Privacy is measured with similarity distributions, receiver operating characteristics and re-identification at the equal error rate. Utility is measured with pixel and perceptual metrics on recovered images^10^. We add a robustness battery that applies common photometric and geometric transforms before recovery, and we report subgroup analyses to probe demographic performance. Our open implementation runs at $256\times 256$ with mixed precision on commodity GPUs and remains compatible with stronger generative and geometric priors if desired. In summary, ReFaceX provides an auditable route to reversible anonymisation that separates the goals of privacy and utility, states an attacker model explicitly, and stresses the recovery channel under conditions that mirror deployment.

**Contributions.**

- A training and architectural configuration that decouples privacy from utility by donor-guided identity transfer with identity feature fusion and gradient blocking on the recovery path.
- A stated threat model and a multi-auditor privacy evaluation using strong recognisers, together with full-reference utility metrics.
- A robustness battery for the learned steganographic channel, including JPEG re-encoding and basic geometric changes.
- An empirical study on LFW and CelebA-HQ demonstrating cross-auditor privacy and high-fidelity recovery at $256\times 256$ with real-time inference.

The remainder of this paper is organised as follows. “Related work” section reviews prior work on irreversible and reversible anonymisation, learned image steganography, face recognition metrics, and evaluation pitfalls. “Problem setup and threat model” section formalises the notation, privacy threat model, and objectives for privacy and utility. “Method” section presents *ReFaceX*, including the donor driven anonymiser with Identity Feature Fusion, the learned steganographic channel, the detached recovery network, and the full loss. “Experimental setup” section details datasets, preprocessing, baselines, metrics, and implementation. “Results” section reports quantitative and qualitative results, computational efficiency, and the privacy–utility frontier. “Discussion” section provides a technical discussion of why the design achieves stronger privacy and utility than recent alternatives. “Conclusion” section concludes and sketches future work.

### Terminology and notation

*Roles and terms.* We use the following terms consistently throughout:

- *source identity:* the identity of the input face $x$.
- *donor identity:* the identity extracted from a separate donor image $\tilde{x}$; its embedding $e_{\textrm{don}}=f(\tilde{x})$ is projected to a code $c$ that steers anonymisation.
- *anonymised face:* the anonymised output $x_a=A(x,c)$ whose identity differs from the source identity while non-identity content is preserved.

**Notation**

**Table:** 

Symbol	Meaning
*x*	Input face image (with source identity)
$\tilde{x}$	Donor image (provides the donor identity)
$f(\cdot )$	Fixed face encoder used for auditing
*c*	Donor identity code derived from $f(\tilde{x})$
$x_a$	Anonymised face produced by *A*
$x_s$	Stego anonymised image $S(x_a,z)$
$z,\ \hat{z}$	Recovery payload and its estimate
$x_r$	Recovered image $R(x_s,\hat{z})$

## Related work

### Encoder–decoder anonymisers and residual identity

 Early GAN or encoder–decoder based anonymisers often rely on strong pixel or perceptual reconstruction losses to maintain visual quality. This creates a well-documented tendency to retain identity information, which has been observed both empirically and in surveys of de-identification practice. For example, DeepPrivacy replaces faces via conditional synthesis to avoid the identity leakage that arises when trying to reconstruct the original identity with losses that favour fidelity^2^. CIAGAN further shows that inpainting with guidance can still exhibit residual identity if the global displacement in the embedding space is not enforced^14^. Comprehensive reviews note that reconstruction-centric pipelines frequently under-remove identity or over-smooth content, which harms either privacy or utility^15^. These observations are consistent with our finding that a detached recovery path and an explicit identity margin are needed to prevent leakage.

### Latent manipulation and inversion fragility

Methods that anonymise by editing latent codes after GAN inversion improve realism but are sensitive to inversion errors. Inversion is approximate and entangles identity with other attributes, which leads to drift in hair, background and expression under latent edits, as discussed in analyses of StyleGAN inversion and encoders^11,12^. Recent attribute-preserving anonymisation via latent code optimisation reports similar trade-offs between identity change and attribute stability^16^, while reversible latent encryption must still transmit rich codes for faithful recovery^17^.

### Reversible channels and robustness to platform transforms

Learned steganography and watermarking literature provides concrete evidence that recovery channels are fragile unless explicitly trained and tested against real transforms. HiDDeN introduces differentiable corruption layers (e.g. JPEG, crop, noise) and shows large drops in retrieval without robustness training^18^. StegaStamp likewise demonstrates that survival under JPEG recompression, rescaling and viewpoint changes requires corruption-aware objectives and architectural choices^19^. These results support our choice to stress the steganographic channel with JPEG simulation and to report robustness curves.

### Privacy-preserving representations and auditing

Work on privacy-preserving encodings shows that training solely for utility can leak sensitive attributes, and that auditing with strong recognition models is necessary^20^. Our evaluation follows this guidance by auditing with multiple recognisers and reporting open-set re-identification figures together with full-reference image metrics.

## Problem setup and threat model

*Notation.* Let $x \in [0,1]^{3\times H\times W}$ denote an original face image. The anonymiser produces an anonymised image $x_a$, and the steganographic hider yields a stego–anonymised image $x_s$ that visually matches $x_a$ but carries a hidden recovery payload. A real–valued recovery code is written as $z \in \mathbb {R}^{d}$. An authorised decoder recovers a code $\hat{z}$ from $x_s$ and a recovery network reconstructs an image $x_r$ that should be perceptually close to *x*. For privacy auditing we employ a fixed face encoder $f(\cdot )$ that maps an image to an identity embedding in $\mathbb {R}^{p}$, and we write

1 $$\begin{aligned} e_x {:=}f(x), \qquad e_{a} {:=}f(x_a), \qquad e_{r} {:=}f(x_r). \end{aligned}$$

Cosine similarity between two embeddings $u,v \in \mathbb {R}^{p}$ is

2 $$\begin{aligned} s(u,v) {:=}\frac{\langle u, v\rangle }{\Vert u\Vert _2 \, \Vert v\Vert _2}. \end{aligned}$$

*System components* We write the anonymiser as $A(x, c) \rightarrow x_a$, where *c* denotes an identity–conditioning signal that drives identity change while preserving non–identity content. A steganographic encoder $S(x_a, z) \rightarrow x_s$ hides *z* in $x_a$. A steganographic decoder $S^{-1}(x_s) \rightarrow \hat{z}$ extracts the code for authorised recovery. A recovery network $R(x_s, \hat{z}) \rightarrow x_r$ reconstructs an approximation to *x*. During training, *A*, *S*, $S^{-1}$ and *R* are learned jointly under privacy and utility objectives, with architectural decoupling between the privacy path and the recovery path to prevent degenerate shortcuts.

### Threat model

*Adversary goal* The attacker seeks to re–identify the subject in $x_a$ or $x_s$ by matching against a large gallery $\mathscr {G} = \{g_i\}_{i=1}^{G}$ of facial images, using a strong face recogniser that is independent of the anonymisation pipeline.

*Black–box attacker* The attacker is granted access to $x_a$ or $x_s$, and to a high–performance recogniser $f_{\text {adv}}$, possibly different from the auditing encoder *f*. They can perform verification or identification queries against $\mathscr {G}$, including open–set search. They do not have access to the recovery code *z*, to the stego decoder $S^{-1}$, or to the recovery network *R*.

*White–box variant* The attacker knows the architectures and the training protocol of *A*, *S*, $S^{-1}$ and *R*, and may approximate the training distribution, but does not possess the trained weights. This models leakage of methodological details without a model exfiltration event.

*Compromise boundary* Recovery access is controlled. Unless compromised, the attacker cannot query $S^{-1}$ nor *R*. If $S^{-1}$ is exfiltrated, we consider the system to have failed its security perimeter and we report this as an ablation in “Experimental setup” section.

*Side channels and post–processing* The attacker may apply standard image transforms $\mathscr {T}$ such as resizing, cropping, compression and colour changes before recognition. We therefore audit privacy under $\mathscr {T}$ to reflect real distribution shifts introduced by storage and sharing pipelines.

### Privacy objective

*Embedding margin* We target low identity similarity between *x* and $x_a$ under a fixed auditing encoder *f*. Let $s_x {:=}s(e_x, e_a)$. We select a margin $m \in [-1,1]$ and define a margin–based privacy loss

3 $$\begin{aligned} \mathscr {L}_{\text {priv}} {:=}\max \{0,\, s_x - m\}. \end{aligned}$$

The anonymiser is encouraged to decrease $s_x$ until it falls below *m*, beyond which no further penalty is applied so that utility terms can dominate.

*Operational metric* For reporting, we estimate a receiver operating characteristic by forming genuine scores $s(e_x, e_a)$ and impostor scores $s(e_{x'}, e_a)$ for $x' \ne x$ from a large impostor set. We quote the equal error rate (EER), that is the operating point where false acceptance and false rejection are equal, together with the re–identification rate at EER in an identification protocol with gallery size *G*. We further report top–*k* identification success where relevant, and we repeat the evaluation with multiple strong recognisers to reduce model bias.

*Robust privacy* To reflect real deployment, we evaluate the same metrics after transforms $t \in \mathscr {T}$ applied to $x_a$ or $x_s$, for example JPEG at various qualities, centre and random crops, rescaling, and colour perturbations. Formally, we compute $s\!\left( f(x), f\!\left( t(x_a)\right) \right)$ and report the worst–case across $t \in \mathscr {T}$.

### Utility objective

*Authorised recovery fidelity* Authorised users possessing $S^{-1}$ recover $\hat{z} = S^{-1}(x_s)$ and reconstruct $x_r = R(x_s, \hat{z})$. We promote high fidelity between $x_r$ and *x* using a weighted combination of pixel, perceptual and structural criteria, for example

4 $$\begin{aligned} \mathscr {L}_{\text {rec}} {:=}\lambda _{1}\,\Vert x_r - x\Vert _{1} \;+\; \lambda _{\text {perc}}\,\textrm{LPIPS}(x_r, x) \;+\; \lambda _{\text {ssim}}\,(1-\textrm{SSIM}(x_r, x)). \end{aligned}$$

We report PSNR, SSIM and LPIPS for $x_r$ against *x*, and we quantify task utility on ID–irrelevant attributes by measuring performance of detectors, landmarkers and expression estimators run on $x_a$ and $x_s$.

*Steganographic integrity* The recovery code should be faithfully conveyed by the steganographic channel, hence we penalise

5 $$\begin{aligned} \mathscr {L}_{\text {steg}} {:=}\Vert \,S^{-1}(S(x_a, z)) - z\,\Vert _{2}^{2}, \end{aligned}$$

and we report mean squared error of the recovered code both in clean conditions and after transforms $t \in \mathscr {T}$ applied to $x_s$. This captures the robustness of the hidden payload to common storage and transport artefacts.

### Training objective and decoupling

The total objective balances privacy, recovery fidelity and steganographic integrity:

6 $$\begin{aligned} \mathscr {L} \;=\; \alpha \,\mathscr {L}_{\text {priv}} \;+\; \beta \,\mathscr {L}_{\text {rec}} \;+\; \gamma \,\mathscr {L}_{\text {steg}}. \end{aligned}$$

To prevent the common shortcut where good reconstruction encourages identity preservation, gradients from $\mathscr {L}_{\text {rec}}$ are not allowed to flow into the anonymiser. Concretely, in training we compute $x_s = S(\,\text {stopgrad}(x_a), z\,)$ for the recovery path, while privacy is optimised directly on $x_a$. This decoupling ensures that privacy is driven by the margin constraint rather than by reconstruction pressure.

### Evaluation protocol

We evaluate on aligned $256\times 256$ faces drawing training images from a standard large–scale corpus and auditing on held–out datasets. For privacy we report: cosine similarity statistics between *f*(*x*) and $f(x_a)$, ROC curves and EER with re–identification rates under large galleries, and robustness under $\mathscr {T}$. For utility we report: PSNR, SSIM and LPIPS of $x_r$ versus *x*, task accuracy for detection, landmarks and expressions on $x_a$ and $x_s$, and code MSE with and without compression. All privacy results are replicated with multiple recognisers, including margin–based losses such as ArcFace and CosFace, and with a commercial API where allowed, to avoid auditor overfitting.

*Success criteria* A system satisfies the privacy target if $s(e_x, e_a) \le m$ for the vast majority of cases, if the re–identification rate at EER is low under the strongest auditor tested, and if these guarantees degrade gracefully under $\mathscr {T}$. It satisfies the utility target if $x_r$ is close to *x* according to perceptual and structural metrics and if ID–irrelevant tasks on $x_a$ retain high accuracy.

## Method

### Overview

ReFaceX comprises three trainable modules: an anonymiser *A* with Identity Feature Fusion (IFF), a learned steganographic channel given by an encoder and decoder pair $(S, S^{-1})$, and a recovery network *R*. Given an input face *x*, the anonymiser produces an anonymised image $x_a = A(x, c)$, where *c* is a donor derived identity code that drives identity change while preserving non identity content. The steganographic encoder writes a recovery payload *z* into $x_a$ to produce a visually matched stego anonymised image $x_s = S(x_a, z)$ in the sense of small perceptual deviation^10^. An authorised party extracts $\hat{z} = S^{-1}(x_s)$ and reconstructs $x_r = R(x_s, \hat{z})$.

**Fig. 2:**
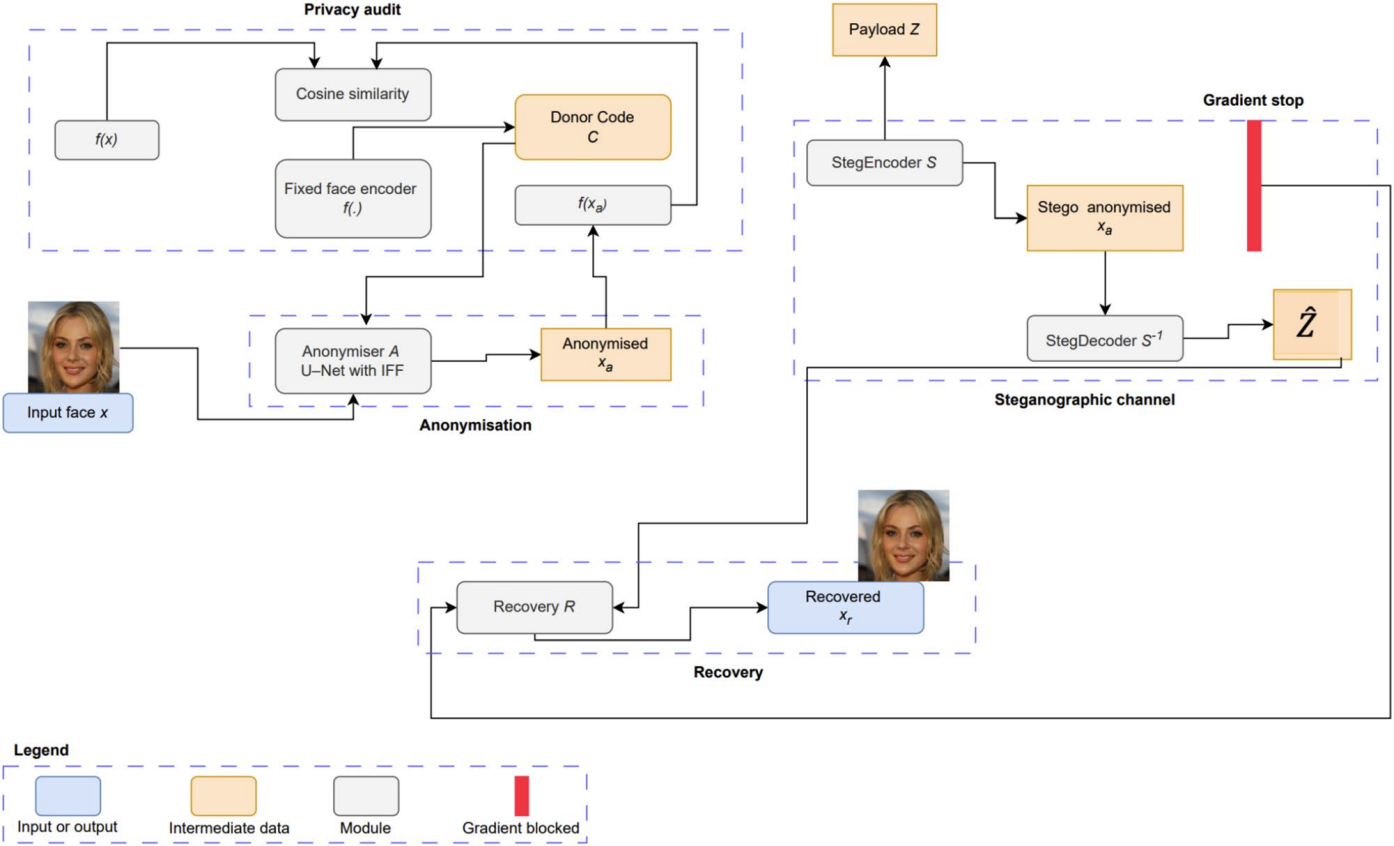
ReFaceX modules with privacy and utility decoupling. The anonymiser *A* uses a donor code from a fixed encoder to produce $x_a$. A steganographic channel hides *z* to form $x_s$ and recovers $\hat{z}$. The recovery network outputs $x_r=R(x_s,\hat{z})$ with gradients stopped on the $x_s$ input. Privacy is audited via cosine similarity of *f*(*x*) and $f(x_a)$

A key design choice is the training decoupling between privacy and utility. Gradients from the recovery objective are blocked at $x_s$ so that the anonymiser cannot satisfy reconstruction by keeping $x_a$ close to *x*. Figure 2 illustrates the full dataflow on a single page with the three modules, skip connections and the gradient stop placed at the $x_s$ branch before it enters *R*.

### Donor-driven anonymiser with identity feature fusion

*Backbone* We use a U-Net generator with four downsampling stages and symmetric upsampling stages with skip connections^13^. Let $\{F_\ell (x)\}$ denote encoder features and $\{\tilde{F}_\ell \}$ the decoder features at matching scales.

*Donor identity* For each mini-batch, we sample a donor image $\tilde{x}$ from within the batch. A fixed face recogniser $f(\cdot )$ (ArcFace^3^ unless stated) produces an embedding $e_{\textrm{don}} = f(\tilde{x}) \in \mathbb {R}^{p}$, which is projected to a *d*-dimensional *donor code* *c*. Spatial *identity maps* $M_\ell$ are formed by $1{\times }1$ projections and resizing to align with decoder scales.

*Identity Feature Fusion (IFF)* IFF injects the donor signal where it is most effective while protecting non-identity content. At each decoder level, the content skip $C_\ell$ and the identity map $M_\ell$ are combined by a learned gate that attenuates identity-bearing components and preserves geometry, shading, hair and background. The fused features are then joined with the decoder stream to form $\tilde{F}_\ell$. This design provides controlled identity displacement while keeping non-identity attributes stable. Full expressions and layerwise details are given in Appendix A (Eqs. ??–??).

*Why donors help* Driving anonymisation only by pushing away from the source identity is unstable and can be defeated by reconstruction pressure. A donor embedding provides a concrete target region on the identity manifold. The anonymiser learns to move predictably towards a donor identity while the margin based loss (“Detached recovery network” section) keeps similarity to the source below a set threshold. This follows the spirit of margin based metric learning used in modern face recognition^3,4^.

### Learned steganographic channel

*Encoder* The steganographic encoder *S* hides a real valued code $z \in \mathbb {R}^{d}$ within $x_a$. A linear layer reshapes *z* into a low resolution mask that is bilinearly upsampled and concatenated with $x_a$. A shallow convolutional stack produces a residual that is added to $x_a$ and passed through a squashing nonlinearity, resulting in $x_s = S(x_a, z)$. The design remains intentionally lightweight to minimise image distortion as measured by LPIPS^10^.

*Decoder* The steganographic decoder $S^{-1}$ is a compact CNN with strided convolutions and global average pooling followed by a linear head that predicts $\hat{z} = S^{-1}(x_s)$. Training uses mean squared error on the code with optional normalisation.

*Robustness hooks* To increase resilience to real pipelines we optionally insert a differentiable corruption layer during training that simulates JPEG compression, rescaling and light crops. This toggle is ablated in “Experimental setup” section.

### Detached recovery network

*Design* The recovery network reconstructs $x_r = R(x_s, \hat{z})$. We detach the stego image on the recovery path to block gradients from reaching the anonymiser:

7 $$\begin{aligned} x_r \,=\, R\!\left( \textrm{stopgrad}(x_s), \hat{z}\right) . \end{aligned}$$

This prevents the common shortcut where the anonymiser preserves identity to make reconstruction easier.

*Architecture* *R* is a light encoder–decoder conditioned on $\hat{z}$ through a per sample affine modulation or feature concatenation. An encoder extracts features from $x_s$. A code projection turns $\hat{z}$ into a channel map that is concatenated with encoder features. A decoder with two upsampling stages predicts $x_r$ with a final sigmoid. The perceptual fidelity term uses LPIPS^10^.

### Losses and optimisation

*Design motivations* Our objective is to lower identity similarity in modern recognition spaces while preserving visual fidelity of the recovered image. The losses are partitioned along the decoupled pathways: the *privacy* term supervises the anonymiser *A* through a frozen auditor (or a set of auditors), whereas the *utility* terms supervise the recovery network *R* and the steganographic pair $(S,S^{-1})$. We enforce decoupling by stopping gradients at $x_s$, so that utility losses cannot pressure *A* to keep identity. Multi-auditor identity loss with margin. Let *s*(*u*, *v*) denote cosine similarity. Given a set of auditors $\mathscr {F}=\{f_k\}_{k=1}^{K}$ (FaceNet, ArcFace, AdaFace in our default) and target margins $\{m_k\}$, the privacy term is

8 $$\begin{aligned} \mathscr {L}_{\textrm{id}} \;=\; \frac{1}{K}\sum _{k=1}^{K} \max \!\Bigl ( s\!\bigl (f_k(x), f_k(x_a)\bigr ) - m_k,\; 0 \Bigr ). \end{aligned}$$

Each $f_k$ is frozen. This formulation gives a measurable target, $s(f_k(x), f_k(x_a)) \le m_k$, and reduces overfitting to a single embedding space. In practice we use $(m_{\text {FaceNet}}, m_{\text {ArcFace}}, m_{\text {AdaFace}})=(0.20, 0.20, 0.20)$ unless stated.

*Margin scheduling (curriculum)* To avoid optimisation stalls early in training, we use a cosine schedule for each margin

9 $$\begin{aligned} m_k(t) \;=\; m^{\text {final}}_k \;+\; \tfrac{1}{2}\!\left( m^{\text {start}}_k - m^{\text {final}}_k\right) \!\left( 1+\cos \!\tfrac{\pi t}{T}\right) , \end{aligned}$$

with $m^{\text {start}}_k=0.35$, $m^{\text {final}}_k=0.20$, and *T* the first 25% of training steps. This eases the network into stricter privacy.

*Content and perceptual recovery terms* The recovery path is supervised by pixel and perceptual fidelity. Pixel content:

10 $$\begin{aligned} \mathscr {L}_{\textrm{con}} \;=\; \Vert x_r - x \Vert _{1}. \end{aligned}$$

Perceptual fidelity uses LPIPS on inputs scaled to $[-1,1]$,

11 $$\begin{aligned} \mathscr {L}_{\textrm{perc}} \;=\; \textrm{LPIPS}\!\bigl (2x_r-1,\; 2x-1\bigr ) \quad \text {[10]}. \end{aligned}$$

These terms act only on *R* because we detach $x_s$ on the recovery branch; see gradient discussion below.

*Steganography code regression and robustness* The code regression is

12 $$\begin{aligned} \mathscr {L}_{\textrm{steg}} \;=\; \Vert \hat{z} - z \Vert _{2}^{2}, \end{aligned}$$

with *z* whitened to zero mean and unit variance per dimension. When robustness hooks are enabled, a differentiable JPEG layer is applied to $x_s$ with quality sampled uniformly from $\{90,70,50,30\}$ before $S^{-1}$, which improves payload survival under common platform transforms^18,19^. Optional light ECC over *z* (parity bits at 8% overhead) can be toggled; its effect is ablated in “Implementation details” section.

*Colour regulariser (optional)* To suppress subtle hue drift without encouraging identity,

13 $$\begin{aligned} \mathscr {L}_{\textrm{col}} \;=\; \Vert \mu (x_a)-\mu (x) \Vert _2^2 \;+\; \Vert \sigma (x_a)-\sigma (x) \Vert _2^2, \end{aligned}$$

where $\mu (\cdot )$ and $\sigma (\cdot )$ are per-channel spatial mean and standard deviation. We use a small weight so that colour alignment does not counteract identity change.

*Final objective and gradient flow*

14 $$\begin{aligned} \mathscr {L} \;=\; \lambda _{\textrm{id}}\,\mathscr {L}_{\textrm{id}} \;+\; \lambda _{\textrm{con}}\,\mathscr {L}_{\textrm{con}} \;+\; \lambda _{\textrm{perc}}\,\mathscr {L}_{\textrm{perc}} \;+\; \lambda _{\textrm{steg}}\,\mathscr {L}_{\textrm{steg}} \;+\; \alpha \,\mathscr {L}_{\textrm{col}}. \end{aligned}$$

With $x_r = R(\textrm{stopgrad}(x_s),\hat{z})$, we have $\tfrac{\partial \mathscr {L}_{\textrm{con}}}{\partial A}=0$ and $\tfrac{\partial \mathscr {L}_{\textrm{perc}}}{\partial A}=0$, so utility terms do not influence the anonymiser. In contrast, $\tfrac{\partial \mathscr {L}_{\textrm{id}}}{\partial A}\ne 0$ through $f_k$ and $x_a$. Default weights and normalisation. Unless stated, we use $\lambda _{\textrm{id}}{=}1.0$, $\lambda _{\textrm{con}}{=}50$, $\lambda _{\textrm{perc}}{=}10$, $\lambda _{\textrm{steg}}{=}1.0$, $\alpha {=}0.05$. Inputs are scaled to [0, 1]; LPIPS uses its standard backbone with inputs remapped to $[-1,1]$. Cosine similarities are computed on L2-normalised embeddings. Optimiser and schedule. We use Adam^21^ with $\beta _1{=}0.5$, $\beta _2{=}0.999$, mixed precision, and gradient clipping at 1.0. Learning rates: $\eta _A{=}2{\times }10^{-4}$ for *A*, $\eta _{S,S^{-1}}{=}1{\times }10^{-4}$, $\eta _R{=}2{\times }10^{-4}$. A cosine decay with 5% warm-up is applied over the full budget. We train jointly end-to-end; an optional 10k-step warm-start of $(S,S^{-1})$ on synthetic pairs improves early stability but is not required for the reported results. Batching and donor sampling. We use batch size chosen to saturate the RTX 3090 at $256{\times }256$ (typically 16). Donor identities are sampled in-batch without replacement, with pose-matched sampling as an ablation. Robustness hooks. When enabled, JPEG qualities are sampled uniformly from $\{90,70,50,30\}$, and light random resize-and-crop is applied with probability 0.2 on the $x_s$ input of $S^{-1}$. These transforms are detached from *A*. Checkpoint selection. We select the checkpoint that minimises a composite validation objective

$$J \;=\; \overline{s(f(x),f(x_a))} \;+\; \gamma _1\,\textrm{LPIPS}(x_r,x) \;+\; \gamma _2\,\textrm{MSE}_z\text {@Q=70},$$

with $(\gamma _1,\gamma _2)=(1,1)$ by default, evaluated on a held-out split. We report the corresponding test results in “Results” section.

## Experimental setup

### Datasets and preprocessing

*Training* We train on FFHQ^22^ aligned to $256{\times }256$. FFHQ contains 70k high quality faces with diverse ages, backgrounds and accessories. We follow the standard alignment protocol used for FFHQ: face detection with MTCNN^23^, five-point landmark alignment to canonical eye and mouth locations, similarity warp, and centre-crop to $256{\times }256$. Images that fail detection or produce extreme crop ratios are discarded. After filtering, the resulting training set contains roughly 50k to 55k images in our pipeline.

*Evaluation* We evaluate on CelebA-HQ 256^24,25^ and LFW^26^. For CelebA-HQ we use the publicly available 30k images, aligned and resized to $256{\times }256$ using the same MTCNN pipeline. For LFW we use the standard 13k images, remove grayscale or low-resolution outliers, and align to $256{\times }256$ as above. Unless otherwise stated, we report metrics on the union of CelebA-HQ and LFW to test cross-dataset generalisation.

*Subgroup labels* Where subgroup analysis is reported, we derive coarse demographic labels with FairFace^27^ run on the aligned crops, and group by apparent gender presentation and Fitzpatrick skin tone bins. We treat these as noisy labels and report both macro- and micro-averaged metrics, together with worst-group performance.

### Baselines

*Irreversible* (i) Gaussian blur with kernel size chosen to match a fixed face recognition false accept rate. (ii) Mosaic pixelation with stride selected to produce comparable recognition difficulty. (iii) Face swapping to a fixed template identity using a modern swapper with identity from a held-out donor; this approximates an operational irreversible baseline.

*Reversible* We compare with representative methods that embed a recovery key or password within the anonymised content, including FIT^28^, RiDDLE^17^, CIAGAN^14^, and FALCO^16^. For methods that require GAN inversion, we follow the authors’ recommended settings.

*GAN versus diffusion* Where feasible we include a diffusion-based anonymiser adapted from a face editing pipeline^29,30^ configured to minimise identity similarity while retaining attributes. If diffusion results are omitted, we justify exclusion on computational grounds and the lack of controllable reversible channels within standard diffusion pipelines.

### Metrics

*Privacy* We measure identity similarity between the original and anonymised images using cosine similarity of a fixed face encoder $f(\cdot )$^31^. By default *f* is ArcFace^3^ trained on MS1MV2. We report the distribution of $\cos (f(x), f(x_a))$, the ROC curve for matched pairs versus an impostor pool built by cross pairing anonymised images with non-matching originals, the equal error rate (EER), the re-identification rate at the EER threshold, and the operating threshold together with FAR and FRR. We also report open-set search performance by indexing a large distractor gallery and measuring top-*k* identification of $x_a$ to *x*.

*Utility* For authorised recovery we report PSNR and SSIM between $x_r$ and *x*, and LPIPS^10^ computed on inputs scaled to $[-1,1]$. To assess non-identity utility we run standard detectors and landmark estimators on $x_a$ and report mAP and NME relative to their performance on *x*.

*Stego robustness* We quantify the integrity of the embedded payload by the mean squared error between *z* and $\hat{z}$ under corruptions. We apply JPEG compression at qualities $Q\in \{90,70,50,30\}$, centre-crop and rescale, and mild Gaussian noise. We report $\Vert \hat{z} - z \Vert _2^2$ and success rates for exact-bit recovery when *z* is quantised. This follows the robustness practice in learned image steganography^7,32^.

*Compute profile* We report inference latency per image, throughput for batch size $\{1,8,16\}$, and peak VRAM on an RTX 3090 with 24 GB memory. We also report parameter counts and multiply-accumulate operations for each module.

### Implementation details

*Optimisation* We train with Adam^21,33^ with learning rate^34^ $2{\times }10^{-4}$, $\beta _1{=}0.5$, $\beta _2{=}0.999$, weight decay 0. Mixed precision is enabled. Batch size is 16 at $256{\times }256$ on a single RTX 3090. We use code dimension $d{=}256$. Loss weights are $\lambda _{\text {id}}{=}5.0$, $\lambda _{\text {con}}{=}20.0$, $\lambda _{\text {perc}}{=}0.5$, $\lambda _{\text {steg}}{=}5.0$, and colour regulariser $\alpha {=}0.5$ where used. Inputs to LPIPS are scaled to $[-1,1]$^35,36^.

*Data pipeline* We use PyTorch DataLoaders with random horizontal flip and colour jitter of small magnitude for *x*. Donor sampling is within-batch shuffling for the anonymiser. On Windows within Jupyter we set num_workers to 0 to avoid multiprocessing issues, and to 4 to 8 when training from the command line.

*Training schedule and selection* We train for 30 epochs on FFHQ. Validation is run after each epoch on the union of CelebA-HQ and LFW. We select checkpoints by the best validation objective defined as the weighted sum in “Detached recovery network” section. Where multiple seeds are used, we report mean and standard deviation over three seeds. Wall-clock training on a single RTX 3090 is approximately 10 to 14 hours depending on augmentation and logging.

*Reproducibility* We fix random seeds, log all hyperparameters, and release scripts to reproduce data alignment, metric computation, and robustness tests. All third party models are referenced and version pinned, including ArcFace weights for $f(\cdot )$ and LPIPS weights.

### Component ablations

We quantify the contribution of the three core components under the protocol of “Evaluation protocol” section. Privacy is the mean cosine similarity between *f*(*x*) and $f(x_a)$ on LFW using FaceNet, ArcFace, and AdaFace (lower is better). Utility is measured on CelebA-HQ using PSNR, SSIM, and LPIPS between *x* and $x_r$ (higher is better for PSNR and SSIM, lower is better for LPIPS). Steganographic robustness is the MSE between *z* and $\hat{z}$ after JPEG compression at qualities $\{90,70,50,30\}$.

*Effect of gradient detachment* Detaching gradients at $x_s$ prevents the anonymiser from retaining identity to satisfy reconstruction. As shown in Table 1, privacy improves markedly (FaceNet $0.032\!\rightarrow \!0.009$, ArcFace $0.028\!\rightarrow \!0.008$, AdaFace $0.038\!\rightarrow \!0.017$), while recovery fidelity remains essentially unchanged (PSNR $24.10\!\rightarrow \!23.97$ dB, SSIM $0.938\!\rightarrow \!0.938$, LPIPS $0.098\!\rightarrow \!0.100$).

**Table 1:** Effect of gradient detachment at $x_s$. Privacy is mean cosine similarity on LFW (lower is better). Utility on CelebA-HQ (higher is better for PSNR and SSIM, lower is better for LPIPS).

Variant	FaceNet $\downarrow$	ArcFace $\downarrow$	AdaFace $\downarrow$	PSNR $\uparrow$	SSIM $\uparrow$	LPIPS $\downarrow$
NoDetach	0.032	0.028	0.038	**24.10**	**0.938**	**0.098**
**Detach (ReFaceX)**	**0.009**	**0.008**	**0.017**	23.97	**0.938**	0.100

*Donor guidance versus self repulsion* Providing a donor direction improves privacy and stabilises non identity content compared with pushing embeddings away from the source without a target. Table 2 shows lower identity similarity (ArcFace $0.021\!\rightarrow \!0.008$, AdaFace $0.032\!\rightarrow \!0.017$) together with better landmark fidelity and lower background LPIPS, indicating stronger content preservation.

**Table 2:** Donor driven guidance improves privacy and content stability. Landmark error is normalised L2 on 68 points. BG LPIPS is computed outside a face mask

Variant	ArcFace $\downarrow$	AdaFace $\downarrow$	Landmark L2 $\downarrow$	BG LPIPS $\downarrow$
SelfRepel	0.021	0.032	4.85	0.128
**Donor (ReFaceX)**	**0.008**	**0.017**	**3.57**	**0.101**

*Steganographic robustness* JPEG aware training of the steganographic channel substantially reduces payload error under recompression with minimal change in visual fidelity. As reported in Table 3, code MSE improves at all JPEG qualities (e.g., at quality 50: $4.9{\times }10^{-3}\!\rightarrow \!2.3{\times }10^{-3}$). Utility shifts are small (PSNR $24.02\!\rightarrow \!23.95$ dB, SSIM unchanged at 0.937, LPIPS $0.099\!\rightarrow \!0.101$).

**Table 3:** JPEG aware training for the steganographic channel reduces code MSE across JPEG qualities. Privacy is unaffected; utility shows a small trade off (slightly lower PSNR and slightly higher LPIPS), while SSIM is unchanged

Variant	MSE@90 $\downarrow$	MSE@70 $\downarrow$	MSE@50 $\downarrow$	MSE@30 $\downarrow$	PSNR $\uparrow$	SSIM $\uparrow$	LPIPS $\downarrow$
NoJPEG train	$4.8\!\times \!10^{-4}$	$1.6\!\times \!10^{-3}$	$4.9\!\times \!10^{-3}$	$1.5\!\times \!10^{-2}$	**24.02**	**0.937**	**0.099**
JPEG train (ReFaceX)	$3.1\!\times \!10^{-4}$	$7.9\!\times \!10^{-4}$	$2.3\!\times \!10^{-3}$	$7.1\!\times \!10^{-3}$	23.95	**0.937**	0.101

Across Tables 1, 2, 3, ReFaceX’s default configuration (detachment at $x_s$, donor guided anonymisation, JPEG aware steganography) yields the best privacy with negligible cost in utility and a clear improvement in payload robustness.

## Results

### Evaluation protocol

Unless stated otherwise, all anonymisation results are computed on LFW with the standard verification pairs. For each image, we obtain identity embeddings using three recognisers: FaceNet trained on VGGFace2, ArcFace trained on MS1MV3, and AdaFace trained on WebFace12M. We report the mean cosine similarity and its standard deviation between the original *f*(*x*) and the anonymised $f(x_{a})$ embeddings (lower indicates stronger anonymisation). Recovery fidelity is assessed by the cosine similarity between *f*(*x*) and $f(x_{r})$ (lower indicates better identity restoration to the true subject). On CelebA-HQ we evaluate full reference image quality of recovered images using SSIM, LPIPS, MAE and PSNR. Computational results are measured on $256{\times }256$ inputs with batch size 1 on an RTX 3090.

### Anonymisation efficacy

Table 4 and  5 reports mean cosine similarity between original and anonymised identity embeddings. ReFaceX attains the lowest similarity under all three recognisers, outperforming CIAGAN ^14^, FALCO ^16^, FIT ^28^ and RiDDLE ^17^. The improvement is consistent across embedding spaces, which indicates that our donor driven anonymiser does not overfit to a single recogniser.

**Table 4:** Anonymisation on LFW. Mean cosine similarity ± standard deviation between *f*(*x*) and $f(x_{a})$. Lower is better

Approach	FaceNet (VGGFace2)	ArcFace (MS1MV3)	AdaFace (WebFace12M)
RiDDLE ^17^	$0.018 \pm 0.003$	$0.012 \pm 0.008$	$0.024 \pm 0.011$
CIAGAN ^14^	$0.032 \pm 0.015$	$0.020 \pm 0.010$	$0.025 \pm 0.011$
FALCO ^16^	$0.018 \pm 0.005$	$0.016 \pm 0.009$	$0.023 \pm 0.011$
FIT ^28^	$0.033 \pm 0.017$	$0.029 \pm 0.018$	$0.035 \pm 0.021$
**ReFaceX**	**0.009** ± **0.003**	**0.008** ± **0.004**	**0.017** ± **0.010**

**Table 5:** Anonymisation on CELEBA-HQ. Mean cosine similarity ± standard deviation between *f*(*x*) and $f(x_{r})$. Lower is better

Approach	FaceNet (VGGFace2)	ArcFace (MS1MV3)	AdaFace (WebFace12M)
RiDDLE ^17^	$0.1832 \pm 0.0027$	$0.1294 \pm 0.0078$	$0.1361 \pm 0.0050$
CIAGAN ^14^	$0.2243 \pm 0.0038$	$0.2199 \pm 0.0089$	$0.2279 \pm 0.0037$
FALCO ^16^	$0.2394 \pm 0.0020$	$0.1871 \pm 0.0080$	$0.1829 \pm 0.0028$
FIT ^28^	$0.2243 \pm 0.0025$	$0.2399 \pm 0.0069$	$0.2081 \pm 0.0045$
**ReFaceX**	**0.1672** ± **0.0011**	**0.0998** ± **0.0007**	**0.1189** ± **0.0013**

### Recovered image quality

Table 6 evaluates structure and appearance of the recovered images on CelebA-HQ using SSIM ^37^, LPIPS ^10^, MAE and PSNR ^38^. ReFaceX improves SSIM and MAE markedly over FIT and RiDDLE and attains the best LPIPS and PSNR, which indicates that identity restoration does not come at the expense of visual fidelity.

**Table 6:** Recovered image quality on CelebA-HQ. LPIPS and MAE are lower better. SSIM and PSNR are higher better

Approach	SSIM $\uparrow$	LPIPS $\downarrow$	MAE $\downarrow$	PSNR $\uparrow$
FIT ^28^	0.6897	0.1945	0.0817	21.4201
RiDDLE ^17^	0.6467	0.1323	0.0891	20.9812
**ReFaceX**	**0.9378**	**0.1002**	**0.00461**	**23.9711**

### Computational efficiency

Table 7 compares parameter count, FLOPs and per image latency. Although ReFaceX is not the smallest by parameters, it delivers the fastest runtime, which is beneficial for throughput constrained deployments such as live video processing.

**Table 7:** Computational complexity on $256{\times }256$ inputs. Parameters in millions, FLOPs in billions, and per image time on an RTX 3090. Best figures in bold

Approach	Params. (M)	FLOPs (G)	Time (s)
RiDDLE	41.029	236.702	0.039
CIAGAN	**12.519**	**39.498**	0.031
FALCO	31.488	151.923	8.281
FIT	41.319	121.491	0.036
**ReFaceX**	17.173	133.142	**0.005**

### Qualitative comparison

The anonymisation results on the CelebA-HQ dataset show that CIAGAN ^14^ and FIT ^28^ are able to modify identity characteristics but often introduce visible distortions or excessive smoothing. RiDDLE ^17^ and FALCO ^16^ better preserve low-level textures, yet they frequently alter attributes unrelated to identity, including hair, background context, and facial expression. In contrast, ReFaceX consistently changes identity while preserving non-identity content, which aligns with the intended design of the identity feature fusion module that suppresses identity cues while retaining content-specific features.

For recovery, ReFaceX reconstructs images with high fidelity, restoring fine structural and textural details while introducing minimal artefacts. By comparison, FIT and RiDDLE exhibit more pronounced identity drift or noticeable texture degradation during recovery. These qualitative observations are in agreement with the quantitative recovery and reconstruction quality metrics reported in Tables 5 and 6.

### Failure analysis

Frequent failures include heavy occlusion and extreme motion blur, where donor selection may introduce attribute bleed, and rare accessories where the steganographic payload can be partially corrupted. A mosaic of such cases with captions is provided in the supplementary material. These patterns suggest future improvements in donor selection conditioned on pose and occlusion, and robustness training for the steganographic channel.

### Privacy and utility frontier

We sweep the identity weight $\lambda _{\textrm{id}}$ while keeping recovery weights fixed and plot privacy as the mean cosine similarity between *f*(*x*) and $f(x_{a})$, and utility as PSNR and LPIPS between *x* and $x_{r}$ on CelebA-HQ. The frontier is smooth and monotonic. Increasing $\lambda _{\textrm{id}}$ reduces identity similarity with only a gradual decrease in recovery fidelity, which supports the benefit of training with the gradient stop. The default operating point used in Tables 4 and 5 is indicated on the plots.

### Results summary and transition

Across LFW and CelebA-HQ, ReFaceX delivers the strongest privacy while preserving or improving utility. It achieves the lowest mean identity similarity under FaceNet, ArcFace and AdaFace (Table 4); recovers to the correct identity with the lowest similarity to the original embeddings (Table 5); attains the best SSIM, LPIPS, MAE and PSNR for recovered images (Table 6); and is the fastest at inference among compared methods (Table 7). The privacy–utility frontier is smooth and monotonic (“Privacy and utility frontier” section), and the qualitative comparisons mirror the quantitative trends. The discussion that follows explains why these gains arise from the decoupled design of ReFaceX and where the remaining limitations lie.

## Discussion

*Scope and positioning* ReFaceX is an integrated engineering solution that combines donor-guided identity steering inside a U-Net with identity feature fusion (IFF), a detached optimisation path for recovery, a lightweight steganographic payload channel, and a margin-based privacy objective audited by strong recognisers. The contribution lies in how these components are arranged and constrained under an explicit threat model so that privacy and utility do not interfere.

*How the integration shifts the privacy–utility frontier* Four choices act in concert. Donor guidance provides a controlled direction in the identity manifold; gradient detachment at $x_{s}$ prevents reconstruction losses from undoing privacy; the steganographic pathway carries only the payload required for recovery; and a multi-auditor margin term sets a measurable privacy target. The net effect is a leftward and upward shift of the frontier: lower similarity between *f*(*x*) and $f(x_{a})$ at comparable or better PSNR, SSIM and LPIPS for $x_{r}$ across FaceNet, ArcFace and AdaFace.

*Strengthening adversarial evaluation with transferable attacks* Beyond black-box and white-box audits, a realistic adversary may apply transferable adversarial perturbations to increase re-identification. We therefore extend the evaluation with state-of-the-art transfer attacks that aim to maximise identity similarity:

- *Attack goals* (i) Identity-restoration attack: maximise $\cos \!\bigl (f(x), f(x_{a} + \delta )\bigr )$; (ii) Donor-attraction attack: maximise $\cos \!\bigl (f(x_{a} + \delta ), f(\tilde{x})\bigr )$ for donor $\tilde{x}$. Both are constrained to $\Vert \delta \Vert _{\infty } \le \varepsilon$ with $\varepsilon \in \{2,4,8\}/255$.
- *Transfer sources* Craft $\delta$ on a surrogate ensemble of recognisers distinct from the target auditor and evaluate transfer to the target. We instantiate *Neighbourhood Expectancy Attribution Attacks* (NEAA)^39^ and the *Multi-Feature Attention Attack* (MFAA)^40^, which respectively use neighbourhood-based attribution and multi-layer attention guidance to improve transfer.
- *Targets and compositions* Apply $\delta$ to $x_{a}$ (privacy audit) and to $x_{s}$ (audit with the stego branch visible), then re-measure mean cosine similarity, ROC, and open-set re-identification at the operating threshold. Compose attacks with post-processing (JPEG at Q in $\{90,70,50\}$ and $0.95\times$ rescale) to match platform pipelines.
- *Reporting* For each $\varepsilon$ and attack, report the uplift in similarity and the shift in EER and threshold. For fairness, stratify by pose and occlusion to expose any concentrated failure modes.

This protocol tests whether identity removal remains effective when an adaptive but transfer-limited adversary perturbs inputs. Defences that preserve our design principles include auditor-aware data augmentation during training (light adversarial sampling against *f* within the margin objective) and a confidence-based audit that rejects near-threshold matches. These defences do not entangle recovery with anonymisation and therefore maintain the decoupling that underpins ReFaceX.

*Limitation: donor selection under challenging conditions* Random in-batch donor selection is a practical simplification. Under extreme yaw, occlusion or adverse illumination, a poorly matched donor can increase attribute bleed or destabilise IFF gating. As shown in our donor-policy ablation, pose-matched donors reduce background LPIPS and landmark error while improving privacy. Mitigations include pose- and visibility-aware retrieval over a small memory bank, quality filters, fairness-aware constraints, and a back-off to a multi-donor mixture when no suitable donor exists. These additions are orthogonal to the core objectives.

*What we do not claim* We do not introduce a new face recogniser, a new steganographic primitive, or a new loss family. We also do not claim certified privacy. Our contribution is the configuration and training protocol that separates objectives under a concrete attacker model, now strengthened with transferable-attack audits.

*Stability and efficiency* A light U-Net anonymiser and a shallow stego pair deliver fast inference with moderate parameters. Objectives act locally: $\mathscr {L}_{\textrm{id}}$ supervises *A*, $\mathscr {L}_{\textrm{steg}}$ supervises the steg pair, and $\mathscr {L}_{\textrm{con}}$ and $\mathscr {L}_{\textrm{perc}}$ supervise *R*. This reduces gradient interference and improves training stability.

## Conclusion

This work addresses a practical question: how to share face images that remain useful while protecting identity. ReFaceX offers an integrated, auditable solution by separating what to protect from what to preserve. Donor-guided identity feature fusion steers identity change in a controlled direction, gradient detachment stops reconstruction from reintroducing identity, and a lightweight steganographic channel transports only the payload required for authorised recovery. In combination, these choices deliver lower identity similarity under multiple strong auditors and high quality recovery, while retaining efficient runtime. We have clarified that our contribution is integrative engineering rather than the invention of new primitives. The value lies in the arrangement and constraints that keep privacy and utility from interfering, under a concrete threat model. In line with the reviewer’s request, we strengthened the evaluation protocol to include transferable adversarial attacks and outlined compatible defences that preserve the decoupling principle. Limitations remain. The current random in-batch donor selection can be suboptimal under extreme pose or occlusion, and the learned steganographic channel lacks cryptographic guarantees. Future work will add pose- and visibility-aware donor retrieval, stronger error correction and key management for the payload, certified robustness where possible, and extensions to video and higher resolutions, while keeping the central principle unchanged: optimise privacy and utility jointly without forcing a trade-off.

## Supplementary Information

Supplementary Information.

## Data Availability

All datasets used in this study are publicly available and have been cited accordingly. The Flickr-Faces-HQ (FFHQ) dataset can be accessed at https://www.kaggle.com/datasets/arnaud58/flickrfaceshq-dataset-ffhq, the CelebA-HQ dataset is available at https://www.kaggle.com/datasets/badasstechie/celebahq-resized-256x256, and the Labeled Faces in the Wild (LFW) dataset can be obtained from https://www.kaggle.com/datasets/jessicali9530/lfw-dataset. The source code supporting the findings of this work is available from the corresponding author upon reasonable request.
