# Supplementary material for: ReFaceX: donor-driven reversible face anonymisation with detached recovery
Source: Sci Rep. 2026 Feb 9;16:7882. doi: 10.1038/s41598-026-39337-2 (PMC12953573; doi:10.1038/s41598-026-39337-2)
Supplement: Supplementary file 1 — Supplementary Information. [file 41598_2026_39337_MOESM1_ESM.pdf]

Appendix A. Identity Feature Fusion (IFF) details

At decoder level ℓ, let C_ℓ be the encoder skip (content) and M_ℓ the donor identity map aligned to the same spatial size. IFF first projects and normalises inputs, then computes a gate and returns an attenuated content feature that is reintroduced into the decoder.

Projections.

$$\hat{C}_\ell = \phi_c(C_\ell), \qquad \hat{M}_\ell = \phi_m(M_\ell), \tag{1}$$

where ϕ_c and ϕ_m are 1×1 convolutions with normalisation and nonlinearity.

Gating.

$$G_\ell = \sigma\big(\psi\big([\hat{C}_\ell \,\|\, \hat{M}_\ell]\big)\big), \tag{2}$$

where ψ is a shallow convolutional stack, $\|$ denotes channel concatenation, and σ is a sigmoid.

$$Z_\ell = \mathrm{SE}\big(\hat{C}_\ell \odot (1 - G_\ell)\big), \qquad \tilde{F}_\ell = \mathrm{Conv}([U_\ell \,\|\, Z_\ell]), \tag{3}$$

where U_ℓ is the upsampled decoder input, $\odot$ is elementwise product, SE is a squeeze–excitation refinement, and Conv is a 3×3 convolutional block.
